# The association between seeking financial compensation and injury recovery following motor vehicle related orthopaedic trauma

**DOI:** 10.1186/s12891-016-1152-2

**Published:** 2016-07-13

**Authors:** Darnel F. Murgatroyd, Ian A. Harris, Yvonne Tran, Ian D. Cameron

**Affiliations:** John Walsh Centre for Rehabilitation Research, The University of Sydney, Kolling Institute, Royal North Shore Hospital, St Leonards, Sydney, NSW 2065 Australia; Ingham Institute for Applied Medical Research and South Western Sydney Local Health District, South Western Sydney Clinical School, UNSW, Sydney, Australia

**Keywords:** Compensation and redress, Injury, Multiple trauma, Outcomes

## Abstract

**Background:**

Motor vehicle related moderate-severe orthopaedic trauma has a major impact on the burden of injury. In Australia, all states and territories provide access to financial compensation following injury in a motor vehicle crash. The aim of this study was to investigate the influence of seeking financial compensation (i.e., making a claim) on injury recovery following motor vehicle related moderate-severe orthopaedic trauma.

**Methods:**

Patients admitted with upper/lower extremity fractures after a motor vehicle crash were recruited from two trauma hospitals. Baseline data were collected in person by written questionnaire within two weeks of injury. Follow up data were collected by a mailed written questionnaire at six, 12 and 24 months. Additional (demographic/injury-related) information was collected from hospital databases, all other measures were self-reported. Outcomes were: Short Form-36 Version 2.0 (SF36v2), Physical/Mental Component Scores (PCS/MCS); Post Traumatic Stress Disorder (PTSD) Checklist Civilian Version (PCL-C); and Global Rating of Change (GRC) scale. Analysis involved descriptive statistics and linear mixed models to examine the effect of compensation status on injury recovery over time.

**Results:**

There were 452 study participants. Baseline characteristics showed: mean age 40 years (17.1 Standard Deviation [SD]); 75 % male; 74 % worked pre-injury; 67 % in excellent-very good pre-injury health; 56 % sustained serious injuries, Injury Severity Score (ISS) 9–15; 61 % had a low-middle range household income.

Overall, after controlling for possible confounders, the compensable group had poorer recovery compared to the non-compensable group for PCS (−2.97 Mean Difference (MD), 95 % CI −4.73, −1.22); MCS (−3.44 MD, 95 % CI −5.62, −1.26); PCL-C (3.42MD, 95 % CI 0.87, 5.99); and GRC (−0.66MD, 95 % CI −1.15, −0.17). Injury recovery over time for all participants showed: PCS improved from 6–12 and 12–24 months; MCS and GRC improved from 6–12 months; and PCL-C did not significantly improve from 6–12 and 12–24 months. Injury recovery over time continued for compensable and non-compensable groups but compensable participants had poorer scores at each time period, especially MCS and PCL-C.

**Conclusions:**

Making a claim was associated with poor injury recovery following motor vehicle related orthopaedic trauma, mainly for mental health. Irrespective of claim status, the majority had poor injury recovery, especially for mental health.

## Background

Orthopaedic trauma is commonly sustained after a motor vehicle crash and often results in hospital admission [1] with many experiencing ongoing pain and physical and psychological disability [2–8]. In addition, motor vehicle related orthopaedic trauma has a major impact on the burden of injury [9, 10].

Analysis of Australian data shows the annual cost of motor vehicle crashes is approximately AU (Australian) $17b or 2.3 % of Gross Domestic Product (GDP) [11]. The greatest economic burden occurs in New South Wales (NSW) where the total cost of motor vehicle crashes is AU$5.7b per annum (in 2003). An evaluation of the Victorian state trauma system reported an increased incidence of hospitalised major trauma and years lived with disability from 2001–2011 [12]. These studies underscore the need for high quality research investigating predictors of recovery following motor vehicle related orthopaedic trauma and demonstrate the substantial economic burden on society.

To date, related research indicates there are numerous predictors of poor injury recovery, the most common being socio-demographic factors such as age, gender, occupation and education. These tend to have conflicting associations, possibly dependent on population differences [4, 6, 7, 13, 14]. Whereas psychosocial factors, for example: high initial pain scores; mental illness; and low self-efficacy, are more consistently associated with poor recovery [2, 5, 6, 13, 15, 16].

For compensation related factors, there is robust evidence from several systematic reviews that seeking financial compensation is associated with poor injury recovery [17–21]. These factors include making a claim [3, 13, 14], seeking legal representation [4, 5, 7, 16], and altering access to financial entitlements [22, 23]. Moreover, qualitative research, which has predominantly focussed on the claims process experience, demonstrates that it can be detrimental to injury recovery, hinder return to work, and be conducive to financial hardship [24–27].

Despite this, the impact of seeking financial compensation remains contentious and the causal relationship is questionable [17, 20, 28, 29]. For example, recent evidence suggests that poor pre-injury mental health status is partly responsible for poor injury recovery in those seeking financial compensation [19, 30]. This is important, particularly with the high prevalence of mental illness in Australia (20 %) [31]. There have been calls for more rigorous research with sound methodology including between and within scheme comparisons in specific populations [28, 29, 32, 33].

All Australian states and territories provide access to financial compensation following injury in a motor vehicle crash and a number of prospective studies have investigated the relationship between compensation related factors and injury recovery. However, these have largely been confined to: mild-moderate injuries [13, 34, 35]; short follow up periods (six months) [2, 4]; and/or studies that include mechanisms of injury other than a motor vehicle crash [3]. This current study followed people with motor vehicle related, moderate to severe orthopaedic trauma in NSW, Australia for two years.

We were primarily interested in exploring the association between claim status and injury recovery. The specific aim was to investigate the influence of seeking financial compensation (i.e., making a claim) on injury recovery following motor vehicle related moderate-severe orthopaedic trauma.

## Methods

### Study design and setting

Patients from two trauma hospitals in Sydney, NSW, were recruited for the inception cohort study between November 2007 and February 2011. These hospitals are two of the seven level one trauma services in NSW (population approximately seven million), and provided a sample of patients that required inpatient hospitalisation following motor vehicle related orthopaedic trauma. Eligible patients were identified through the hospital database, and then invited to participate. Where possible, an English speaking family member was used to interpret for patients from from Culturally and Linguistically Diverse (CALD) backgrounds (i.e., spoke a language other than English at home) [36].

Inclusion criteria were:

- admission to hospital within two weeks of injury;
- involvement in a motor vehicle crash;
- age 18 years or over; and
- one or more upper or lower extremity fracture (humerus, radius, ulna, pelvis, acetabulum, femur, patella, tibia, fibula, talus, calcaneus).

Exclusion criteria were:

- dementia or a significant pre-existing cognitive impairment preventing the ability to consent;
- spinal cord injury;
- Glasgow Coma Score <12 on admission;
- amputation of a limb; or
- isolated phalangeal, carpal, metacarpal, tarsal or metatarsal fractures.

There were 32 variables, and allowing for a minimum of 10 participants per variable, a sample size of 320 was required for sufficient statistical power for regression analysis. Comparable research indicated that a final sample size of 450 was required to allow for a possible 25 % loss to follow up [4, 37, 38]. However, based on power calculations for repeated measures in linear mixed models used in this study, a sample size of n greater than 100 was required in order to achieve power greater than 0.9 [39]. Questionnaires were mailed for follow up at six, 12 and 24 months. Up to six attempts to contact participants were made by telephone and/or by mailed questionnaire if no response was received within three weeks.

Within two weeks post-injury, baseline data were recorded in person by written questionnaire. Hospital databases were used to collect additional demographic and injury related information. All other measures were self-reported. The selection of study factors was based on similar research with relevance to the study aims [5–8, 40]. Approval for the study was given by the governing human research ethics committees (South Western Sydney Local Health District, South Eastern Sydney Local Health District, and The University of Sydney).

### Injury related factors

The Abbreviated Injury Scale (AIS) (1990 Revision, Update 98) was used to code all injuries [41]. The scale has an injury ranking system from one to six (six is not survivable). Algorithms were used to calculate the Injury Severity Score (ISS) and New Injury Severity Score (NISS): sums of the squares of the three highest AIS scores from different body regions (ISS), and irrespective of body region (NISS). They indicate potential mortality [42]. Classifications for injuries were minor-moderate [1–8], serious [9–15] or severe-critical [16–75].

### Socio-demographic factors

A number of socio-demographic factors were measured such as age, gender, marital status, occupation, and education. Household income measurements were exclusive and inclusive of household structure, this allowed for any potential difference in income distribution [44]. Current measures for Return To Work (RTW) are not standardised, therefore, RTW was self-reported and included duration (full-time/part-time) and level of work (full/modified duties) [45].

### Health related factors

For an indication of baseline health status a number of self-reported chronic illnesses were included: asthma; cancer; heart and circulatory conditions; diabetes; arthritis; osteoporosis; mental and behavioural problems; and neck/back disorders. The National Health Priority Areas initiative lists these conditions as inflicting significant social and financial costs within Australia [46]. The definition of a chronic condition was taken from the Australian Bureau of Statistics (ABS) Health Survey, it is one which a patient currently has, and it has lasted or is expected to last for six months or more [44, 46]. Additional measures were: recent injuries (other than the motor vehicle crash) that required medical attention in the last four weeks or a decreased usual activity; medication use in the last two weeks for a chronic illness; and smoker status [44].

The definitions and categories of other self-reported factors such as: recovery expectations for work and usual activities; risk of long/short term harm due to alcohol consumption; Body Mass Index (BMI); and health status are documented in the Tables. Additional information about study factors, outcomes and methodology including predictors of RTW can be found in another publication by the same authors [47].

### Compensation related measures

In NSW, there is a privately underwritten, modified common law scheme which provides Compulsory Third Party (CTP) personal injury insurance. To travel on a public road all motor vehicles need to be registered and insured for CTP. An injured person claims against the owner or driver of the vehicle at fault. From April 2010, anyone injured in a motor vehicle crash (irrespective of fault) can claim restricted entitlements of medical expenses and lost wages up to AU$5,000. For Workers Compensation (WC), a publically underwritten scheme exists that is managed by private insurers. An injured person can claim following a motor vehicle crash that happened whilst travelling between the worksite, home and/or any work-related place (irrespective of fault). In addition, notification of an injury must occur within 48 h [48, 49]. In 2015, the NSW government scheme regulators amalgamated forming the State Insurance Regulatory Authority (SIRA).

In each scheme, claims need to be submitted within six months of injury. Insurers have three months to decide whether to accept or deny liability for the claim. To allow early payment of medical expenses or weekly wage benefits (for WC), insurers can accept provisional liability [49]. For CTP, insurers can pay lost wages for financial hardship, but decisions are made case-by-case. Other joint entitlements for past and future losses include medical expenses, lost income, and pain and suffering/impairment [48, 49]. For both schemes, people can seek legal representation at any time.

Self-reported compensation related measures of crash on a public road and at fault were taken at baseline. Whereas, making a claim (Yes/No) was measured by patient interview at six months because the majority of participants would not have been able to answer this question within two weeks of injury.

### Health status outcome measures

General health status was measured using the Short Form-36 Version 2.0 (Australia) (SF36v2). This self-report instrument encompasses physical and mental health and measures an individual’s own perception of their health status across eight domains (physical functioning, role-physical, bodily pain, general health, vitality, social functioning, role-emotional and mental health). The scores range from 0–100 with higher scores representing better health status. The Physical and Mental Component Scores (PCS/MCS) are summary scores of the eight domains [50]. The SF36 has high test-retest reliability, content validity and construct validity [50]. The minimal clinically important difference of PCS/MCS scores ranges from 2 to 7 for different diseases; 5 was selected as it is a commonly used threshold [51, 52]. The SF36v2 has been widely used in trauma populations [3, 4, 13, 14, 34, 35].

Post-Traumatic Stress Disorder (PTSD) was selected as an outcome measure because it is commonly associated with motor vehicle related orthopaedic trauma [21]. PTSD was measured using the PTSD Checklist Civilian Version (PCL-C): a self-report 17 item checklist of symptoms. Scores range from 1–5 (not at all – extremely) indicating at what level participants were bothered by a symptom over the past month [53]. Total scores range from 17–85. A cut-off score of 44 (i.e., ≥44 have PTSD) is recommended for overall diagnostic efficiency for people injured in a motor vehicle crash [54]. The checklist has been tested for reliability and validity, and it can be used for a provisional clinical diagnosis [54, 55]. A structured clinical interview would be required for confirmation. Evidence suggests 5 points is the minimum threshold to report clinical change [56]. The words ‘stressful experience’ was replaced with ‘accident’ to tailor the questionnaire to the motor vehicle crash [53].

A Global Rating of Change (GRC) scale is designed to quantify improvement or deterioration over time following an intervention or to monitor the course of a condition. These scales are often used in conjunction with more specific measures such as those encompassing pain, disability and quality of life [57]. GRC scales have high face validity and allow a person to rate their recovery in terms of what is important to them [58]. For this scale, participants were asked ‘how do you rate your health now, compared to your usual level of health prior to the accident?’ A recommended 11 point scale was used, ranging from −5 – 5 (−5 = vastly worse, 0 = unchanged, 5 = completely recovered) with a minimal clinically important difference of 2 points [59].

### Data analysis

Descriptive statistics were used to summarise baseline characteristics and ANOVA and chi-squared tests were used to determine baseline characteristics by claim status at six months. The variables met the assumptions of independence, homoscedasticity and normality.

Linear mixed models, which expand the general linear model and account for the dependency between repeated measurements collected for each participant across time, were used to examine the effect of making a claim on injury recovery over time. The fixed effects were claim status, Index of Relative Socioeconomic Disadvantage (IRSD), gender, ISS, education, language other than English, BMI, vehicle type, risk of short term harm due to alcohol consumption, self-reported at fault, pre-morbid neck pain in the last six months, crash on a public road, self-assessed pre-injury health status, and time. The co-variate was age and the interaction tested was claim status by time. These variables were selected based on level of interest (i.e., hypothesis driven) from past research [4–8], and significant confounding variables at baseline for claim status with a *p*-value <0.1. Other significantly different baseline variables between the two claim status groups not included in the model were work hours before injury and pre-injury job satisfaction because they were measures only related to those working pre-injury, and alcohol use in the past year which is a construct of risk of long term harm due to alcohol consumption. Using the model estimates, marginal means and standard errors were reported for each health status measure (SF36v2 PCS/MCS, PCL-C and GRC) at six, 12 and 24 months.

To assess the impact of attrition bias, sensitivity analysis was conducted using the ‘per protocol’ sample. This sample was selected based on participation for all measurement time points (i.e., six, 12 and 24 months). To assess the impact of pre-existing mental health problems, sensitivity analysis was conducted on the sample without those who reported pre-existing mental health problems (*n* = 19). All data analysis was performed using SPSS statistical software version 22 (SPSS Inc, USA).

## Results

**Fig. 1:**
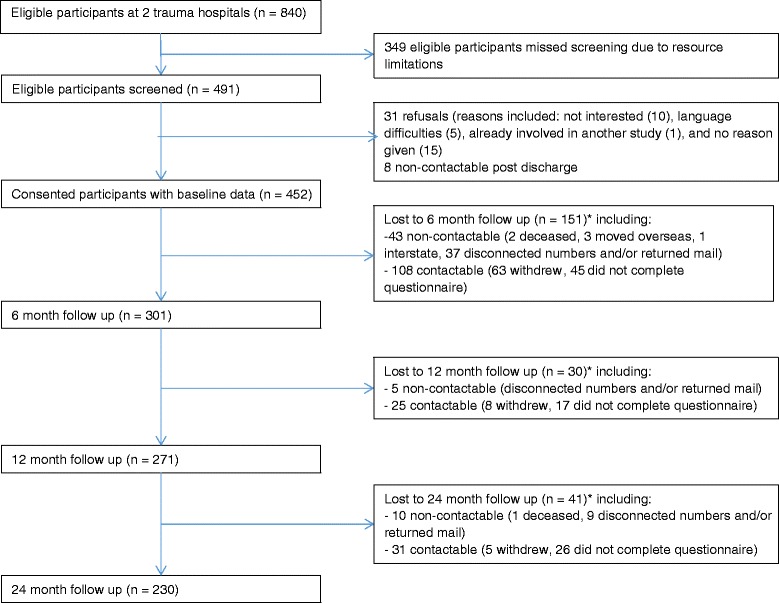
Flow chart of study participants

From November 2007 to February 2011, 840 eligible participants were admitted to hospital across both sites, 491 were screened, and 452 (92 %) consented to participate. There were 349 eligible participants that were not screened due to resource limitations. There were 31 refusals and eight who were discharged and unable to be contacted. Additional information about recruitment and follow up for all study participants is shown in Fig. 1.

### Baseline characteristics

The baseline characteristics of all 452 participants showed the mean age was 40 years (17.1 SD), range 18–87 years. Serious injuries with an ISS/NISS of 9–15 were sustained by 56 % (ISS) and 42 % (NISS) respectively. The majority were male (75 %) and 59 % were in middle and lower household income brackets, placing them in the middle and lower two quintiles of the Index of Relative Socioeconomic Disadvantage (IRSD). Only 17 % had obtained a bachelor degree or above and the majority of participants were in trades, clerical, service, transport, or labouring occupations. Just over one-third (37 %) spoke a language other than English at home. At the time of injury 74 % worked, the majority full time (83 %), on full duties (96 %). Job satisfaction was high (96 %) and 90 % expected to return to work following injury. Only 35 % self-reported at fault in the crash and 91 % of crashes occurred on a public road.

Excellent-very good pre-injury health was perceived by 67 %, good health by 26 %, and fair-poor by 7 %. For other health factors: 35 % had a chronic illness; 60 % were overweight or obese; 27 % had taken medication in the last two weeks; and 28 % were current smokers. Overall, the majority (93 %) had a low risk of long term harm due to alcohol consumption but a larger risk (56 %) of short term harm (i.e., risk of alcohol-related injury).

There were significant differences in pre-injury/baseline characteristics between those who made a claim (at six months) and those who did not, regardless of whether the claim was accepted by the insurer. Of note, those with greater eligibility to make a claim under NSW legislation did so (i.e., self-reported not at fault and crash on a public road). There were no significant differences in pre-injury/baseline health status measures between those who made a claim and those who did not. However, these measures largely related to physical health. These results are illustrated in Table 1. Of the 301 (67 %) participants who completed the six month follow up questionnaire, 294 answered the compensation related questions and of those 61 % (179/294) made a claim. Subsequent results are based on this subset (294) of participants.

**Table 1:** Baseline characteristics and health status by claim status at six months

Variable	No.	Claim made (*n* = 179)	No claim made (*n* = 115)	*P*
Age (years), Mean (SD)	294	41.3 (16.0)	39.7 (16.7)	0.42
Injury Severity Score, No. (%)				0.62
Minor - moderate 1-8	294	44 (24.6)	31 (27.0)	
Serious 9-15		105 (58.7)	61 (53.0)	
Severe - critical 16-75		30 (16.8)	23 (20.0)	
New Injury Severity Score, No. (%)	294			0.49
Minor- moderate 1-8		34 (19.0)	25 (21.7)	
Serious 9-15		67 (37.4)	48 (41.7)	
Severe - critical 16-75		78 (43.6)	42 (36.5)	
Index of Relative Socioeconomic Disadvantage (IRSD)^a^, Mean (SD)	294	969 (149)	990 (149)	0.23
Index of Relative Socioeconomic Disadvantage (IRSD)^a^, No. (%)	294			0.09
Most disadvantaged		53 (29.6)	28 (24.3)	
Disadvantaged		17 (9.5)	6 (5.2)	
Average		37 (20.7)	17 (14.8)	
Advantaged		37 (20.7)	38 (33.0)	
Most advantaged		35 (19.5)	26 (22.6)	
Male, No. (%)	294	120 (67.0)	90 (78.3)	0.04
Marital status, No. (%)	293			0.34
Single		58 (32.4)	46 (40.4)	
Married/de facto		103 (57.5)	56 (49.1)	
Divorced/widowed/separated		18 (10.1)	12 (10.5)	
Education skill level^b^, No. (%)	291			0.06
Bachelor degree and above		36 (20.1)	17 (15.0)	
Certificate and advanced diploma		66 (36.9)	53 (46.9)	
Secondary education		65 (36.3)	42 (37.2)	
Pre-primary and primary education		11 (6.1)	1 (0.9)	
Occupation skill level^b^, No. (%)	294			0.24
Home duties/retired		15 (8.4)	6 (5.2)	
Managers/administrators/ professionals/associate professionals		39 (21.8)	31 (27.0)	
Tradespersons/advanced clerical and service workers		50 (27.9)	42 (36.5)	
Intermediate clerical/sale/service production/transport workers		28 (15.6)	14 (12.2)	
Elementary clerical/sales/service/labourers/related workers		47 (26.3)	22 (19.1)	
Work status before injury (working), No. (%)	292	140 (78.2)	91 (80.5)	0.64
Work level before injury (full duties), No. (%)	231	133 (95.0)	89 (97.8)	0.28
Work hours before injury^c^ (full time), No. (%)	227	105 (76.8)	80 (88.9)	0.02
Pre-injury job satisfaction^d^ (satisfied), No. (%)	231	136 (97.1)	84 (92.3)	0.09
Recovery expectations for work^e^ (yes), No. (%)	229	125 (89.9)	85 (94.4)	0.23
Recovery expectations for usual activities^e^ (days), No. (%)	278			0.37
≤90		104 (60.5)	74 (69.8)	
91-180		37 (21.5)	20 (18.9)	
181-365		24 (14)	10 (9.4)	
≥366		7 (4.1)	2 (1.9)	
Language other than English (yes), No. (%)	294	72 (40.0)	32 (27.8)	0.03
Total yearly household income^f^ (before tax, AUD) excluding number of people in household, No. (%)	270			0.47
≤$39,999		42 (25.5)	22 (21.0)	
$40,000-$79,999		55 (33.3)	32 (30.5)	
≥$80,000		68 (41.2)	51 (48.6)	
Total adjusted yearly household income^f^ (before tax, AUD) including number of people in household, No. (%)	270			0.32
≤$39,999		97 (58.8)	58 (55.2)	
$40,000-$79,999		54 (32.7)	32 (30.5)	
≥$80,000		14 (8.5)	15 (14.3)	
Body Mass Index (BMI)^g^ (kg/m^2^), No. (%)	292			0.07
<18.50 (underweight)		4 (2.3)	3 (2.6)	
18.50-24.99 (normal)		49 (27.7)	47 (40.9)	
≥25.00 (overweight)		78 (44.1)	35 (30.4)	
≥30.00 (obese)		46 (26.0)	30 (26.1)	
Smoking history, No. (%)	293			0.23
Current smoker		34 (19.1)	28 (24.3)	
Ex-smoker		47 (26.4)	36 (31.3)	
Never smoked		97 (54.5)	51 (44.3)	
Self-reported chronic illnesses (yes), No. (%)	294	71 (39.7)	37 (32.2)	0.19
Medication use (current), No. (%)	293	52 (29.2)	32 (27.8)	0.80
*Recent injury other than* crash (yes), No. (%)	292	7 (3.9)	5 (4.4)	0.85
Alcohol use in the past year^g^, No. (%)	294			0.23
Never		37 (20.7)	18 (15.7)	
≤1/month		45 (25.1)	20 (17.4)	
2-4 times/month		42 (23.5)	30 (26.1)	
2-3 times/week		32 (17.9)	24 (20.9)	
≥4 times/week		23 (12.8)	23 (20.0)	
Alcohol use in the past year^h^ (standard drinks^i^ on a typical day when you were drinking), Median (Min.- Max.)	293	2.0 (0.0-33.0)	3.0 (0.0-35.0)	0.05
Alcohol use in the past year^h^ (≥6 standard drinks^i^/occasion), No. (%)	294			0.15
Never		84 (46.9)	39 (33.9)	
Less than monthly		47 (26.3)	34 (29.6)	
Monthly		16 (8.9)	14 (12.2)	
Weekly		27 (15.1)	20 (17.4)	
Daily or almost daily		5 (2.8)	8 (7.0)	
Risk of long term harm due to alcohol consumption^j^(standard drinks^i^/week), No. (%)	293			0.17
Low risk - ≤28 male or ≤14 female		172 (96.1)	104 (91.2)	
Risky - 29–42 male or 15–28 female		4 (2.2)	4 (3.5)	
High risk - ≥43 male or ≥29 female		3 (1.7)	6 (5.3)	
Risk of short term harm due to alcohol consumption^j^ (yes), No. (%)	294	95 (53.1)	76 (66.1)	0.03
Self-reported at fault (yes), No. (%)	293	33 (18.5)	72 (62.6)	<0.001
Vehicle type, No. (%)	294			0.02
Motor vehicle		111 (62.0)	52 (45.2)	
Motorcycle		61 (34.1)	58 (50.4)	
Bicycle		7 (3.9)	5 (4.3)	
Pre-morbid neck pain in last 6 months (yes), No. (%)	294	6 (3.4)	9 (7.8)	0.09
Post-morbid neck pain (yes), No. (%)	294	38 (21.2)	21 (18.3)	0.54
Crash on a public road (yes), No. (%)	294	170 (95.0)	96 (83.5)	0.001
Self-assessed pre-injury health status^k^, No. (%)	294			0.06
Excellent		55 (30.7)	35 (30.4)	
Very good		72 (40.2)	41 (35.7)	
Good		46 (25.7)	26 (22.6)	
Fair-Poor		6 (3.4)	13 (11.3)	

^a^The Index of Relative Socioeconomic Disadvantage (IRSD) is a summary measure of economic and social conditions within a particular area/postcode (e.g., employment, fluency in English and household size). It is taken from the Census of Population and Housing: Socio-Economic Indexes for Areas (SEIFA), Cat no. 2039.0.55.001: Australian Bureau of Statistics; 2001. A low score is indicative of greater socioeconomic disadvantage

^b^Measures for occupation and education are from the Australian Standard Classification of Occupations (ASCO), Cat. No. 1220.0, Australian Bureau of Statistics 1997 and the Australian Standard Classification of Education (ASCED), Cat. No. 1272.0, Australian Bureau of Statistics 2001

^c^Measures for full-time (usually working at least 35 h per week) and part-time (usually working 1–35 h per week) are from the Australian Health Survey: Users' Guide, 2011–13, Cat. No. 4363.0.55.001, Australian Bureau of Statistics [45]

^d^Pre-injury job satisfaction is based on the stem question from the Measure of Job Satisfaction questionnaire by Traynor, M. and Wade, B. 1993

^e^Recovery expectations was based on two measures from a large Canadian study of injured workers with soft tissue injuries by Cole et al. (2002) due to the lack of validated measures

^f^Categories of income are from the Household, Income and Labour Dynamics in Australia (HILDA) Survey Wave 6 Household Questionnaire. Adjusted household income divides household income by the sum of points: 1 for the first person ≥15 years; 0.5 for each additional person ≥15 years; and 0.3 for each person <15 years. This is taken from the National Health Survey: Users’ Guide, Cat.no. 4363.0.55.001. Australian Bureau of Statistics 2004–05

^g^BMI classification is from the Global Database on Body Mass Index, World Health Organisation

^h^Questions are from the Alcohol Use Disorders Identification Test: Self-Report Version (AUDIT-C) from the Drink-less program, The University of Sydney. http://sydney.edu.au/medicine/addiction/drinkless/resources.php

^i^1 standard drink contains 12.5 ml or 10 g of alcohol according to the National Health and Medical Research Council (NHMRC), Australian Alcohol Guidelines Health Risks and Benefits, October 2001

^j^Risk of long and/or short term harm due to alcohol consumption was assessed with the National Health and Medical Research Council (NHMRC) levels

^k^Self-assessed pre-injury health status is based on Question 1 from the Short Form 36, Version 2.0, (SF36v2)

For loss to follow up, there were significant differences between responders and non-responders at each period. The results for six, 12 and 24 months are shown in Table 2. Consistently, at six, 12 and 24 months, non-responders were younger and more likely to have smoked or not to have worked pre-injury. For other variables there was no significant difference (*p* > 0.05) between responders and non-responders (data not shown).

**Table 2:** Baseline characteristics and health status of participants in the study compared to non-participants at six, 12 and 24 month follow up

	Participation at six months			Participation at 12 months			Participation at 24 months		
Variable	Yes^a^ (*n* = 301)	No (*n* = 151)	*P*	Yes^a^ (*n* = 271)	No (*n* = 181)	*P*	Yes^a^ (*n* = 230)	No (*n* = 222)	*p*
Age (years), Mean (SD)	41.2 (16.5)	36.5 (17.8)	0.006	41.8 (16.9)	36.5 (16.8)	0.001	42.7 (16.6)	36.5 (17.1)	<0.001
Marital status, No. (%)			0.001			0.068			0.002
Single	107 (35.7)	80 (53.7)		100 (37.3)	87 (48.1)		77 (33.9)	110 (49.5)	
Married/defacto	162 (54.0)	54 (36.2)		140 (52.2)	76 (42.0)		127 (55.9)	89 (40.1)	
Divorced/widowed	31 (10.3)	15 (10.1)		28 (10.4)	18 (9.9)		23 (10.1)	23 (10.4)	
Occupation skill level^b^, No. (%)			0.029			0.180			0.023
Home duties/retired	22 (7.3)	17 (11.3)		22 (8.1)	17 (9.4)		19 (8.3)	20 (9.0)	
Managers/professionals	71 (23.6)	27 (17.9)		66 (24.4)	32 (17.7)		60 (26.1)	38 (17.1)	
Tradespersons	93 (30.9)	33 (21.9)		81 (29.9)	45 (24.9)		71 (30.9)	55 (24.8)	
Intermediate clerical	43 (14.3)	21 (13.9)		35 (12.9)	29 (16.0)		27 (11.7)	37 (16.7)	
Elementary related	72 (23.9)	53 (35.1)		67 (24.7)	58 (32.0)		53 (23.0)	72 (32.4)	
Work status before injury (working), No. (%)	233 (77.9)	101 (66.9)	0.011	210 (78.1)	124 (68.5)	0.023	182 (79.9)	152 (68.5)	0.006
Total yearly household income^c^No. (%)			0.018			0.080			0.153
≤$39,999	68 (24.5)	47 (35.1)		64 (25.3)	51 (32.3)		52 (24.1)	63 (32.3)	
$40,000-$79,999	89 (32.1)	47 (35.1)		80 (31.6)	56 (35.4)		73 (33.8)	63 (32.3)	
≥$80,000	120 (43.3)	40 (29.9)		109 (43.1)	51 (32.3)		91 (42.1)	69 (35.4)	
Smoking history, No. (%)			<0.001			0.020			0.008
Current smoker	64 (21.3)	61 (40.7)		62 (23.0)	63 (35.0)		49 (21.4)	76 (34.4)	
Ex-smoker	86 (28.7)	33 (22.0)		76 (28.1)	43 (23.9)		68 (29.7)	51 (23.1)	
Never smoked	150 (50.0)	56 (37.3)		132 (48.9)	74 (41.1)		112 (48.9)	94 (42.5)	
Medication use (current), No. (%)	89 (29.7)	32 (21.2)	0.055	85 (31.5)	36 (19.9)	0.006	73 (31.9)	48 (21.6)	0.014
Recovery expectations for usual activities^d^ (days), No. (%)			0.072			0.899			0.367
≤90	184 (64.4)	83 (59.7)		155 (60.5)	112 (66.7)		131 (60.4)	136 (65.7)	
91-180	58 (20.4)	36 (25.9)		58 (22.7)	36 (21.4)		49 (22.6)	45 (21.7)	
181-365	34 (11.9)	11 (7.9)		34 (13.3)	11 (6.5)		31 (14.3)	14 (6.8)	
≥366	9 (3.2)	9 (6.5)		9 (3.5)	9 (5.4)		6 (2.8)	12 (5.8)	
Vehicle type, No. (%)			0.002			0.006			0.005
Motor vehicle	169 (56.1)	102 (67.5)		155 (57.2)	116 (64.1)		129 (56.1)	142 (64.0)	
Motorcycle	120 (39.9)	37 (24.5)		107 (39.5)	50 (27.6)		94 (40.9)	63 (28.4)	
Bicycle	12 (4.0)	12 (7.9)		9 (3.3)	15 (8.3)		7 (3.0)	17 (7.7)	

^a^Participation status ‘yes’ was measured using the information recorded in variables - work status at six, 12 and 24 months and the SF36, Physical Component Score (PCS) at six, 12 and 24 months respectively

** *P* < 0.01, *P < 0.05, *NS* not significant

^b^The measure for occupation is from the Australian Standard Classification of Occupations (ASCO), Cat. No. 1220.0, Australian Bureau of Statistics 1997. See Table 1, Occupational skill level for all categories

^c^Categories of income are from the Household, Income and Labour Dynamics in Australia (HILDA) Survey Wave 6 Household Questionnaire. Income is before tax (AUD) and excluding number of people in household

^d^Recovery expectations was based on two measures from a large Canadian study of injured workers with soft tissue injuries by Cole et al. (2002) due to the lack of validated measures

### Influence of claim status on injury recovery over time

The association between claim status and injury recovery over time are shown in Tables 3, 4 and 5. Table 3 – the mean differences in injury recovery scores between the two groups, Table 4 – the association between time and injury recovery, and Table 5 – the mean injury recovery scores between the claim status groups over time (interaction).

**Table 3:** Association of claim status and injury recovery measures using linear mixed model analyses^a^

Health status measure	Mean difference (SE)	95 % CI	*p*-value
SF-36v2 PCS^b^	−2.97 (0.89)	−4.73, −1.22	0.001
SF-36v2 MCS^b^	−3.44 (1.11)	−5.62, −1.26	0.002
PCL-C^c^	3.42 (1.31)	0.87, 5.99	0.009
GRC^b^	−0.66 (0.25)	−1.15, −0.17	0.009

^a^Adjusted for age, gender, ISS, IRSD, education skill level, language other than English, BMI, risk of short term harm due to alcohol consumption, self-reported at fault, vehicle type, pre-morbid neck pain in the last 6 months, crash on a public road, self-assessed pre-injury health status, time and claim status by time

^b^A negative mean difference indicates that the compensable group had on average a poorer outcome where higher scores indicate better outcomes. Health status measures are: Short Form-36 Version 2.0 Physical Component Score (SF-36v2 PCS); Short Form-36 Version 2.0 Mental Component Score (SF-36v2 MCS); and Global Rating of Change (GRC) scale

^c^A positive mean difference indicates that the compensable group had on average a poorer outcome where higher scores indicate poorer outcomes. Health status measure is: PTSD Checklist – Civilian Version (PCL-C)

**Table 4:** Association of time, measured from 6–12 months and 12–24 months after injury, and injury recovery measures, using linear mixed model analyses^a^

Health status measure	6-12 months			12-24 months		
	Mean difference (SE)	95 % CI	*p*	Mean difference (SE)	95 % CI	*p*
SF-36v2 PCS^b^	−2.38 (0.88)	−4.11, 0.64	0.007	−2.62 (0.96)	−4.51, −0.73	0.007
SF-36v2 MCS^b^	−2.32 (1.10)	−4.48, −0.16	0.032	−1.88 (1.19)	−4.22, 0.47	0.116
PCL-C^c^	2.06 (1.30)	−0.50, 4.61	0.114	2.10 (1.41)	−0.66, 4.86	0.136
GRC^b^	−0.74 (0.25)	−1.23, −0.26	0.003	−0.22 (0.27)	−0.75, 0.31	0.415

^a^Adjusted for age, gender, ISS, IRSD, education skill level, language other than English, BMI, risk of short term harm due to alcohol consumption, self-reported at fault, vehicle type, pre-morbid neck pain in the last 6 months, crash on a public road, self-assessed pre-injury health status, time and claim status by time

^b^A negative mean difference indicates improvement over time where higher scores indicate better outcomes. Health status measures are: Short Form-36 Version 2.0 Physical Component Score (SF-36v2 PCS); Short Form-36 Version 2.0 Mental Component Score (SF-36v2 MCS); and Global Rating of Change (GRC) scale

^c^A positive mean difference indicates improvement over time where higher scores indicate poorer outcomes. Health status measure is: PTSD Checklist – Civilian Version (PCL-C)

**Table 5:** Injury recovery measures^a^ by claim status at 6, 12 and 24 months after injury

Health status measure	No compensation claim		Compensation claim	
	Mean (SE)	95 % CI	Mean (SE)	95 % CI
SF36v2-PCS^b^				
6 months	42.88 (1.77)	39.40, 46.36	40.90 (1.73)	37.51, 44.28
12 months	46.04 (1.87)	42.37, 49.71	42.50 (1.77)	39.03, 45.97
24 months	48.59 (1.92)	44.82, 52.36	45.19 (1.81)	41.64, 48.74
SF36v2-MCS^b^				
6 months	40.70 (2.00)	36.77, 44.63	34.01 (2.14)	29.80, 38.21
12 months	42.17 (2.12)	38.01, 46.34	37.24 (2.18)	32.95, 41.53
24 months	43.12 (2.17)	38.86, 47.39	40.06 (2.24)	35.66, 44.45
PCL-C^b^				
6 months	39.91 (2.57)	34.87, 44.95	44.01 (2.51)	39.09, 48.92
12 months	38.12 (2.71)	32.80, 43.43	41.68 (2.56)	36.66, 46.70
24 months	36.50 (2.79)	31.03, 41.97	39.10 (2.62)	33.96, 44.25
GRC^b^				
6 months	−1.27 (0.50)	−2.25, −0.30	−2.00 (0.48)	−2.95, −1.05
12 months	−0.52 (0.53)	−1.55, 0.51	−1.26 (0.49)	−2.23, −0.29
24 months	−0.42 (0.54)	−1.48, 0.64	−0.93 (0.51)	−1.92, 0.07

^a^Marginal means based on linear fixed effect model with time and claim status as fixed effects. Adjusted for age, gender, ISS, IRSD, education skill level, language other than English, BMI, risk of short term harm due to alcohol consumption, self-reported at fault, vehicle type, pre-morbid neck pain in the last 6 months, crash on a public road, self-assessed pre-injury health status, time and claim status by time

^b^Health status measures are: Short Form-36 Version 2.0 Physical Component Score (SF-36v2 PCS); Short Form-36 Version 2.0 Mental Component Score (SF-36v2 MCS); PTSD Checklist – Civilian Version (PCL-C); and Global Rating of Change (GRC) scale

Table 3 showed that for each measure (PCS, MCS, PCL-C and GRC) the compensable group had poorer recovery than the non-compensable group at the three time periods. However, although these differences were statistically significant; they may be of marginal clinical importance taking into account the minimal clinically important difference for each measure (i.e., 5 points for PCS, MCS and PCL-C, and 2 points for GRC). The greatest differences in scores between the two groups were seen in mental health (MCS and PCL-C).

Table 4 showed that the association between time and injury recovery differed depending on the measure used: for PCS, participants improved from 6–12 and 12–24 months; for MCS and GRC, participants improved from 6–12 months only; and for PCL-C participants did not significantly improve from 6–12 or 12–24 months. Although these changes were statistically significant, they appeared to be of marginal clinical importance.

Table 5 looked at the differences in injury recovery over time between the compensable and non-compensable groups, results indicated that both groups improved, but compensable participants had poorer scores compared to the non-compensable participants at each time period. The differences were greatest when looking at MCS and PCL-C scores. The interaction effect between time and claim status was not significant, that is: the comparative rate of recovery between compensable and non-compensable groups was not dependent on time.

Lastly, there was no significant difference in all injury recovery measures at six, 12 and 24 months for participants who were at fault in a crash before and after 1 April 2010 (when the CTP scheme changed). To assess the impact of attrition bias, the sensitivity analysis showed that those lost to follow up had no significant greater likelihood of delayed recovery on all measures (PCS, MCS, PCL-C, GRC) compared to those who remained in the study. This was based on the similar mean difference scores over time, at six, 12 and 24 months (data not shown). To assess the impact of pre-existing mental health problems (*n* = 19), the sensitivity analysis showed that those with pre-existing mental health problems had no significant greater likelihood of delayed recovery on all measures (PCS, MCS, PCL-C, GRC) compared to those without pre-existing mental problems (data not shown).

## Discussion

In this study, those who made a claim had poorer injury recovery than those who did not, the greatest difference being in mental health scores (MCS and PCL-C). Overall, regardless of claim status, injury recovery continued over time for most measures (PCS, MCS, GRC). For PTSD (PCL-C) there was no significant improvement. Many statistically significant differences in physical and mental health scores between compensable and non-compensable groups may be of marginal clinical importance.

### Influence of claim status on injury recovery over time

Our study reinforces existing research showing that seeking financial compensation is associated with poor injury recovery; this has been demonstrated across different jurisdictions and study populations [17–21]. Current evidence suggests that seeking financial compensation is associated with poor injury recovery for two reasons: firstly, the characteristics and circumstances of those who pursue a claim; and secondly, the claims process. These are not mutually exclusive and are likely to be co-dependent.

It is posited that those seeking financial compensation have poor pre-existing health status, for example: mental health problems [19]; vulnerability to stress [30]; and/or higher rates of obesity [60]. In our study, there were no differences in pre-injury/baseline health between those who made a claim and those who did not. However, this should be interpreted cautiously as the measures largely encompassed physical and not mental health, and the greatest differences between the two groups post-injury were related to mental health (MCS and PCL-C). Given the prevalence of mental illness (population prevalence 20 %) and related conditions such as chronic pain (population prevalence 17–20 %) in Australia [31, 61], it is probable that a significant number of people who made a claim had pre-existing mental health problems. Participants were asked at baseline about pre-existing mental health problems, but specific diagnostic tools were not used.

With respect to other circumstances, those eligible to claim did so, that is: self-reported not at fault and crash on a public road. Fault status was taken into account but other granular measures such as blame, external attributions of responsibility and/or a sense of perceived injustice were not. Previous studies have shown that these factors were associated with: increased pain intensity [2, 62]; greater rates of PTSD [63, 64]; and depression post injury [65]. Such factors are multi-dimensional (e.g., perceived injustice focusses on severity/irreparability of loss and blame/unfairness) and could have contributed to poor recovery particularly for those who self-reported as not at fault [62].

The second point relates to the claims process, which qualitative research has found to be: detrimental to injury recovery; conducive to financial hardship; and tied to stigmatisation of injured workers [24–27]. These themes prevail across different study populations and jurisdictions. Notably for moderate-severe injuries [25, 26], there is much at stake: access to financial entitlements for treatment, non-economic (pain and suffering) and economic loss; and/or assistance with return to work. Unsurprisingly, people find this stressful and it can have a substantial impact on their mental health [17, 19, 21, 30].

Furthermore, it has been proposed that seeking financial compensation is a consistent predictor of PTSD due to: the stressful claims process; constant reminders of the motor vehicle crash; and rumination over crash circumstances and ongoing symptoms (e.g., at medico-legal assessments, with treating health professionals and insurers) [21]. Taking into account the greater PTSD symptomatology and poorer mental health status in the compensable group, it is plausible this could be due to any one or more of these factors.

Notwithstanding the impact of claim status on injury recovery, there appeared to be only marginal improvements in physical and mental health measures over time in both groups, albeit less in the compensable group. Despite abundant research into predictors of recovery following moderate-severe orthopaedic trauma, many of which are unrelated to injury severity [2, 4–7, 14–16], it remains of concern that a population of mostly young working age males with (self-reported) excellent-good pre-injury health do not recover to physical and mental health population norms two years after injury. These results have been replicated elsewhere [5–8, 66].

### Strengths and limitations

This prospective study was a large cohort of moderate-severe injuries following motor vehicle related orthopaedic trauma. Standardised and validated measures were used; these were based on existing research including large population studies [5–8, 40]. Follow up was repeated at three intervals: six, 12 and 24 months.

Additional baseline measures would have been advantageous including: initial pain intensity; mental health co-morbidities such as anxiety, depression and other affective disorders; and social support indices. These factors have been associated with seeking financial compensation and poorer outcomes following orthopaedic trauma [2, 5–7, 13, 15, 16, 21, 31]. Many baseline health measures were self-reported, which has been associated with underestimating the prevalence of risk factors in the general population [67]. This could have impacted our results, although attempts were made to mitigate this by collecting baseline data in person, and the construct of simple questions with clear parameters to enhance recall of information.

Other limitations were participant recruitment solely from hospitals, a moderate number of unscreened eligible participants, and moderate loss to follow up. For the unscreened eligible participants, they were similar in injury type/severity and mechanism of injury to the screened eligible participants. Further, recruitment was conducted over a sustained timeframe (2007–2011) to meet the sample size. For loss to follow up, the study population characteristics are a plausible reason for this, participants were predominantly younger males who tended to be of lower socioeconomic status and who worked in semi-unskilled occupations. They were often contactable but would not return questionnaires (see Fig. 1). Additional sensitivity analysis showed this did not impact our results. Future research may benefit from a larger sample size and more resources allocated to recruitment and follow up particularly for a study with a similar population and aims.

### Future research and policy implications

There are considerable implications for planning future rehabilitation services for this population. Irrespective of claim status, many have ongoing physical and mental health problems that do not resolve post injury. In Australia, rehabilitation is largely directed towards older people (average age 74 years, 58 % female) [68]. Moreover, the current focus is on emergency and surgical care [69]. Our findings, supported by other research, demonstrate that this population could benefit from additional services [5, 7, 8, 66].

Over one in five Australians experience mental illness but only one third of these people seek treatment [31]. There is a greater prevalence of mental illness in young people and males are less likely than females to seek treatment. Further, of the two-thirds who do not seek treatment, 90 % report not needing it [31]. This indicates that younger males who are more likely to sustain motor vehicle related orthopaedic trauma have a greater risk of mental health problems post injury, and not recovering, and not seeking treatment even if it was available.

There is a need to trial interventions in this population. Self-management programs are a viable avenue, particularly those with psychosocial components [70, 71]. Internet delivered therapy for chronic pain and anxiety disorders has shown promising results [72, 73]. There is also substantial evidence of efficacy for medication use and cognitive behavioural therapy for mental illness [74, 75]. The challenge will be attracting people to treatment without attrition and identifying possible barriers [31, 71].

In terms of seeking financial compensation, our findings indicate that if eligible, those with moderate-severe injuries are likely to make a claim and have poor injury recovery. There are numerous tools to conduct risk assessments, especially for co-morbidities [2, 5, 13, 16, 40], but less guidance for approval of appropriate treatment. However, in NSW insurers are bound by legislation, and financial entitlements exist for injuries that are causally related to the motor vehicle crash [48, 49]. Examples include: treating an exacerbation of major depression, not the entire illness; or providing a vocational program for return to part-time work in the presence of capacity for full-time work. For the injured person, clinicians and insurers, this delineation can be confusing, difficult to sustain and costly. Furthermore, it does nothing to establish mutual trust or build positive relationships between parties [24, 26, 27]. If desired, legislative change may be the only way to address this issue.

Alternatively, recommendations from qualitative research could alleviate other adversarial and stressful aspects of the claims process by: redesigning procedures for medico-legal assessments; reducing onerous paperwork; improving communication between the parties; using internet-based technology; making timely decisions about entitlements; encouraging early access to treatment; and providing incentives to return to work [24–27, 30]. These initiatives could diffuse some of the negativity associated with seeking financial compensation and improve injury recovery.

Lastly, instruments including perceived injustice, blame, and/or attributions of external responsibility could be advantageous in future studies when investigating the impact of the seeking financial compensation on injury recovery [62, 76]. Previous mixed methodology research attests to the importance of these factors [2, 24, 26, 27, 62, 65].

## Conclusions

Making a claim following motor vehicle related orthopaedic trauma was associated with poor injury recovery, mainly in relation to mental health status and PTSD. However, this may be of marginal clinical importance. Irrespective of claim status, the majority had poor injury recovery on all measures over time, especially for mental health problems. These findings lend credence to existing research and bring into focus the need for efficacious mental health interventions. The reasons why seeking financial compensation is associated with poor injury recovery remains complex. There is a need for initiatives to manage potential co-morbidities and address the adversarial aspects of scheme design.

## Abbreviations

ABS, Australian Bureau of Statistics; AIS, abbreviated injury scale; ASCED, Australian standard classification of education; ASCO, Australian Standard Classification of Occupations; AU, Australian dollar; AUDIT-C, alcohol use disorders identification test: self-report version; BMI, body mass index; CALD, culturally and linguistically diverse; CI, confidence interval; CTP, compulsory third party; GDP, gross domestic product; GRC, global rating of change; HILDA, household, income and labour dynamics in Australia; IRSD, index of relative socioeconomic disadvantage; ISS, injury severity score; MCS, mental component score; MD, mean difference; NHMRC, national health and medical research council; NISS, new injury severity score; NSW, New South Wales; PCL-C, PTSD checklist civilian version; PCS, physical component score; PTSD, post traumatic stress disorder; RTW, return to work; SD, standard deviation; SEIFA, socio-economic indexes for areas; SF36v2, short form-36 version 2.0; SIRA, state insurance regulatory authority; WC, workers compensation
